# Mild traumatic brain injury-induced persistent blood–brain barrier disruption is prevented by cyclosporine A treatment in hypertension

**DOI:** 10.3389/fneur.2023.1252796

**Published:** 2023-11-20

**Authors:** Dominika Lendvai-Emmert, Zsofia Dina Magyar-Sumegi, Emoke Hegedus, Nikolett Szarka, Balint Fazekas, Krisztina Amrein, Endre Czeiter, Andras Buki, Zoltan Ungvari, Peter Toth

**Affiliations:** ^1^Department of Neurosurgery, Medical School, University of Pecs, Pecs, Hungary; ^2^Neurotrauma Research Group, Szentagothai Research Centre, University of Pecs, Pecs, Hungary; ^3^Department of Anaesthesiology and Intensive Therapy, Medical School, University of Pecs, Pecs, Hungary; ^4^Department of Primary Health Care, Medical School, University of Pecs, Pecs, Hungary; ^5^ELKH-PTE Clinical Neuroscience MR Research Group, University of Pecs, Pecs, Hungary; ^6^Department of Neurosurgery, Faculty of Medicine and Health, Orebro University, Orebro, Sweden; ^7^Department of Neurosurgery, Vascular Cognitive Impairment and Neurodegeneration Program, Oklahoma Center for Geroscience and Healthy Brain Aging, University of Oklahoma Health Sciences Center, Oklahoma City, OK, United States; ^8^Department of Public Health, International Training Program in Geroscience, Doctoral School of Basic and Translational Medicine, Semmelweis University, Budapest, Hungary

**Keywords:** mTBI, hypertension, BBB, CyPA, neuroinflammation

## Abstract

**Introduction:**

Mild traumatic brain injury (mTBI) and hypertension synergize to induce persistent disruption of the blood–brain barrier (BBB), neuroinflammation and cognitive decline. However, the underlying mechanisms are not known. Cerebral production of Cyclophilin A (CyPA) is induced in hypertension and after TBI, and it was demonstrated to activate the nuclear factor-κB (NF-kB)- matrix-metalloproteinase-9 (MMP-9) pathway in cerebral vessels leading to BBB disruption.

**Methods:**

To test the role of CyPA in mTBI- and hypertension-induced BBB disruption we induced mTBI in normotensive and spontaneously hypertensive rats (SHR), then the animals were treated with cyclosporine A (a specific inhibitor of CyPA production) or vehicle for 7 days. We assessed BBB permeability and integrity, cerebral expression and activity of the CyPA-NF-kB-MMP-9 pathway, extravasation of fibrin and neuroinflammation.

**Results:**

We found that mild TBI induced BBB disruption and upregulation of the CyPA-NF-kB-MMP-9 pathway in hypertension, which were prevented by blocking CyPA. Cyclosporine treatment and preservation of BBB function prevented accumulation of blood-derived fibrin in the brain parenchyma of hypertensive rats after mTBI and reversed increased neuroinflammation.

**Discussion:**

We propose that mTBI and hypertension interact to promote BBB disruption via the CyPA-NF-kB-MMP-9 pathway, and inhibition of cyclophilin production after mTBI may exert neuroprotection and improve cognitive function in hypertensive patients.

## 1 Introduction

Mild traumatic brain injury (mTBI) is the most frequent form of head trauma, affecting specifically young athletes and the elderly population, who are prone to orthostatic hypotension and to fall (1). Importantly, pre-existing comorbid medical conditions exacerbate TBI-induced pathophysiological changes (2). The most frequently identified comorbid condition affecting mTBI patients is hypertension (3, 4). Accordingly, we have shown earlier that single mTBI induced persistent, long-term disruption of the blood–brain barrier (BBB) in hypertensive rats, leading to persistent accumulation of blood borne substances in the brain parenchyma, neuroinflammation and cognitive decline (5). However, the underlying mechanisms have not been established, yet.

Cyclophilin A (CyPA), an intracellular and secretable chaperone protein of the immunophilin family, was shown to be increased in the sera of patients after severe TBI (6) and was also demonstrated to be a biomarker for untreated hypertension (7). Importantly, cyclophilin levels were increased in isolated brain microvessels of rats after severe controlled cortical impact and inhibiting the production and function of CyPA by a non-toxic dose of cyclosporine A significantly attenuated BBB disruption induced by the trauma (8). Bell et al. provided evidence that cyclophilin A activates the nuclear factor-κB (NF-kB)-matrix-metalloproteinase-9 (MMP-9) pathway in pericytes, which then leads to disintegration of tight junctions and disruption of the BBB. They showed that apolipoprotein E 3 (ApoE3) secreted from astrocytes suppressed the NF-kB-MMP-9 pathway in pericytes by inhibiting CyPA via the Low-density lipoprotein receptor–related protein 1 (LRP1) receptor, and that lack of such suppression, along with activation of ApoE4 led to enhanced expression and function of NF-kB and MMP-9. BBB disruption preceded decreased cortical activity and reduction in neuritic density and presynaptic and postsynaptic proteins in these animals (9). Supporting the role of the ApoE-CyPA-NF-kB-MMP-9 pathway in TBI Teng et al. showed that severe brain trauma of ApoE3 knock out mice induced the activation of the NF-kB and MMP-9, leading to BBB disruption, increased brain water content, and impaired motor function of the animals (10). Based on these we tested the hypothesis that in hypertensive rats mild traumatic brain injury induces activation and upregulation of the CyPA-NF-kB-MMP-9 pathway, which contributes to persistent disruption of the blood–brain barrier, accumulation of blood borne substances in the brain parenchyma and neuroinflammation.

## 2 Methods

All procedures were approved by the Animal Welfare Committee, University of Pecs, and the National Scientific Ethical Committee on Animal Experimentation, Hungary (nr: BA02/2000-35/2022) and carried out in accordance with the ARRIVE guidelines.

### 2.1 Mild traumatic brain injury in normotensive and hypertensive rats

Spontaneously hypertensive (SHR, male, *n* = 16, Janvier Labs, Le Genest Saint Isle, Mayenne, France) and Wistar Han (*n* = 16, Toxi-Coop Zrt. Budapest, Hungary) male rats (300–350 g, 12–15 weeks of age) were studied. Animals were kept in the animal housing facility of the Szentagothai Research Centre (University of Pecs, Hungary) in specific pathogen-free conditions and were fed *ad libitum* throughout the experimental period. Experimental mild TBI was induced with the utilization of an impact acceleration weight-drop model, originally published by Marmarou et al. (11). Anesthesia was induced in an induction box with 5% isoflurane (Baxter, Deerfield, IL, United States) in a 70:30 N2:O2 gas mixture. Once the anesthesia stabilized, the animals’ head was fixed in a stereotaxic frame. From this point, the anesthesia was carried out in 2% isoflurane in the same gas mixture. After removing the hair from the animals’ scalp, a midline incision was performed and the periosteum on the top of the skull was removed. In the midline, halfway between the exposed bregma and lambda sutures, a stainless-steel disc was fixed directly to the bone with cyanoacrylate glue. Then the animal was laid on a foam bed in a prone’ position. The helmet was positioned centrally under the weight-leading plexiglass tube. Experimental mild TBI was induced by dropping the 450 g brass weight from 25 cm height. No skull fractures were observed. After TBI the helmet was removed, and the surgical area was disinfected, and the wound was sutured.

During the surgical procedure, the physiologic parameters of the animals were monitored by a pulse oximeter (MouseOx Plus, Starr Life Sciences Corp., Oakmont, PA, United States), body temperature was monitored by Homeotermic Monitoring System (Harvard Apparatus, Holliston, MA, United States) and maintained with a heating pad at 37°C.

At the pre-determined survival time—on the 8th day post-injury rats were anesthetized [with i.p. administration of 200 mg/kg body weight sodium thiopental (Thiopental 500 mg Powder for Solution for Injection, Sandoz, Sandoz AG, Basel, Switzerland)] and transcardially perfused with physiological saline. Blood samples were collected directly by puncturing the heart of the animals before the perfusion. As the perfusion was completed the brains were removed, weighed, and homogenized [with the utilization of Omni Bead Ruptor Elite bead mill homogenizer (Omni International Inc., Kennesaw GA, United States)] in phosphate-buffered saline (PBS) solution in a 1:9 ratio. Subsequently the homogenates were divided for further measurements.

### 2.2 Pharmacological inhibition of cyclophilin A and blood pressure measurements

After the induction of mTBI, both SHR and Wistar rats were divided randomly into 4 groups, each group containing 8 animals. One group from each strain (8–8 animals) received Cyclosporin A treatment. Sandimmun Concentrate (50 mg/mL, Novartis International AG, Basel, Switzerland) was diluted with physiological saline to reach a 10 mg/mL Cyclosporin A solution. This solution was administered intraperitoneally 15 min after the mTBI in a 20 mg/kg body weight dosage and from the next day in 10 mg/kg/day for 6 days (12). The control groups (8–8 SHR and Wistar animals) received the same amount of physiological saline intraperitoneally as the cyclosporine A treated group at the same time points for 7 days. Blood pressure of the animals was measured with a Hatteras SC1000 non-invasive, tail-cuff blood pressure analysis system (Hatteras Instruments Inc., Cary, NC, United States) three times: 1 day before mild TBI, on the first day post-injury as well as on the 8th day post-injury.

### 2.3 Blood–brain barrier disruption

On the 8th day after the mTBI (24 h after the last i.p. treatment)—2 h before the perfusion—2% Evans blue dye (Sigma, Burlington, MA, United States) was administered intraperitoneally (7 mL/kg) to each animal for measuring the integrity of the blood–brain barrier. Brain homogenate samples were centrifuged (12,000 rpm for 20 min), and absorbance of supernatants were measured at 620 nm with a BMG Labtech Clariostar microplate reader (BMG Labtech GmbH, Ortenberg, Germany).

### 2.4 ELISA and MMP-9 activity

Cyclophilin A (CyPA), Matrix Metalloproteinase-9 (MMP-9), apolipoprotein E (ApoE), Nuclear factor kappa B (NF-kB), Claudin-5 (CLDN5), Occludin (OCLN) as well as Tight junction protein-1 (Zonula occludens-1; ZO-1) were measured from brain homogenates. Commercially available ELISA kits were ordered from Wuhan Fine Biotech Co. Ltd. Wuhan, China and MyBioSource Inc. San Diego, CA, United States (catalog numbers: ER0889, ER0139, ER0353, MBS722386, MBS7249154, MBS025670, MBS2603759). Throughout all the ELISA measurement processes the instructions manuals of the kits were strictly followed. Optical density (OD) was measured at 450 nm with a BMG Labtech Clariostar microplate reader (BMG Labtech GmbH, Ortenberg, Germany). In case of the brain homogenate samples, the protein concentration, expressed as ng or pg./ml can be considered as the protein content in 100 mg wet weight brain tissue.

MMP-9 activity in the brain homogenate samples was measured by the utilization of the SensoLyte® Plus 520 MMP-9 Assay Kit Fluorimetric and Enhanced Selectivity (Cat. No. AS-72017; AnaSpec Inc., Fermont, CA, United States) excluding the application of the monoclonal anti human MMP-9 antibody (coated to the microplate). After 2 h incubation of the samples with the MMP-9 substrate, 5-FAM/QXL™520 FRET peptide (Ex/Em = 490 nm/520 nm upon cleavage) at room temperature, fluorescence intensity (excitation at 490 nm, emission at 520 nm) was measured by a BMG Labtech Clariostar microplate reader (BMG Labtech GmbH, Ortenberg, Germany).

### 2.5 Western blotting

To remove DNA, the homogenates were centrifuged at 35,000 × g on 4°C for 20 min. To detect the protein concentration, the light absorption of the supernatants was measured by spectrophotometry on 595 nm (Lowry’s method, Detergent Compatible Protein Assay Kit, Bio-Rad, Hercules, CA, United States). Aliquots containing 50 μg of protein were mixed with 4× Laemmli buffer (25 mL 1 M Tris–HCl, pH 6.8, 40 mL glycerol, 8 g SDS, 10 mL 100 mM EDTA, 10 mL 100 mM EGTA and 1 mL 1% bromophenol blue brought up to 100 mL with distilled water) and denatured by boiling for 5 min. The proteins were separated based on their size in SDS-containing 10% polyacrylamide gel. The gels were electro-blotted onto PVDF membranes (Hybond-P, GE Healthcare, United Kingdom) with a BioRad Trans-Blot® Turbo™ Transfer System.

The non-specific binding sites were blocked in 5% nonfat dry milk dissolved in TBS-Tween (10 mM Tris-base, 150 mM NaCl, 0.2% Tween-20, pH 8.0). Primary antibodies (Fibrin GTX19079 GeneTex, Irvine, CA, United States) were added, diluted in 3% BSA-TBS-Tween solution, and incubated overnight. Unbound antibodies were removed by washing the membranes five times in TBS-Tween. Then the membranes were incubated with secondary anti-mouse antibodies conjugated with horseradish-peroxidase [HRP; Invitrogen Goat anti-Mouse IgG/IgM (H + L) Secondary Antibody] diluted 1:2,000 in 5% nonfat dry milk blocking solution. After washing the membranes five times in TBS-Tween, the chemiluminescent signal (Immobilon Western, Millipore Corporation, Billerica, MA, United States) was detected using the G:Box gel documentation system (Syngene, Cambridge, United Kingdom). To remove the antibodies the blots were stripped and reprobed with ß-actin antibody (Cell Signaling Technology, 1:2,000) to check the equal amount of loaded proteins.

### 2.6 Quantitative real-time RT-PCR

RNA extraction from each brain sample (*n* = 8, in each group) was performed by applying NucleoZole reagent (Macherey-Nagel GmbH, Duren, Germany) according to the manufacturer’s instructions. Isolated RNA samples were re-suspended in nuclease-free water, quantified at 260 nm with NanoDrop and stored at −80°C. Before cDNA synthesis, DNase digestion (Amplification Grade DNase I; Sigma-Aldrich) was implemented (25°C for 15 min and 72°C for 10 min) to reach greater RNA purity.

Subsequently, cDNA was constructed from DNase-digested total RNA in 20 μL reactions with High Capacity cDNA Reverse Transcription Kit (Thermo Scientific) applying random primers according to the manufacturer’s guidelines. cDNA samples were stored at −20°C and used as a template in real-time PCR reactions.

Gene expression was measured in duplicates by real-time PCR using SensiFast SYBR Green (BioLine, London, United Kingdom) with an ABI Prism 7500 instrument (Applied Biosystems). The following primers were applied as before (5), IL-1β, forward: 5′-GAG TCT GCA CAG TTC CCC AA-3′, reverse: 5′-ATG TCC CGA CCA TTG CTG TT-3′; IL-6, forward: 5′-CAC AAG TCC GGA GAG GAG AC-3′, reverse: 5′-GCC ATT GCA CAA CTC TTT TCT CA-3′; TNFα, forward: 5′-CAG CAA CTC CAG AAC ACC CT-3′, reverse: 5′-GGA GGG AGA TGT GTT GCC TC-3′; β-actin, forward: 5′-GTA ACC CGT TGA ACC CCA TT-3′, reverse: 5′-CCA TCC AAT CGG TAG TAG CG-3′ supplemented with nf NF-kB, forward: 5′-ACC TGG AGC AAG CCA TTA GC-3′ and reverse: 5′ -CGG ACC GCA TTC AAG TCA TA-3′.

The amplification profile started at 95°C for 10 min, which followed 40 cycles of 15 s at 95°C, 30 s at 57°C, and finally 1 min at 72°C. Quantitative measurements were normalized to β-actin mRNA level as a housekeeping gene (13, 14).

### 2.7 Statistical analysis

Data were analyzed with GraphPad Prism 8.4.3 software (GraphPad Software LLC., San Diego, CA, United States), by analysis of variance (ANOVA). A *p*-value < 0.05 was considered statistically significant. Data are expressed as mean ± S.E.M.

The ImageJ software (National Institutes of Health, United States) was used for densitometry. The presented photos are from a series of experiments with similar results. Densitometric values are the mean + S.D. for the indicated repeat number of independent experiments. Significance of differences was determined using one-way ANOVA testing applying Tukey corrections for multiple samples.

## 3 Results

### 3.1 The role of cyclophilin a in mild traumatic brain injury-induced persistent disruption of the blood–brain barrier in hypertensive rats

**Figure 1:**
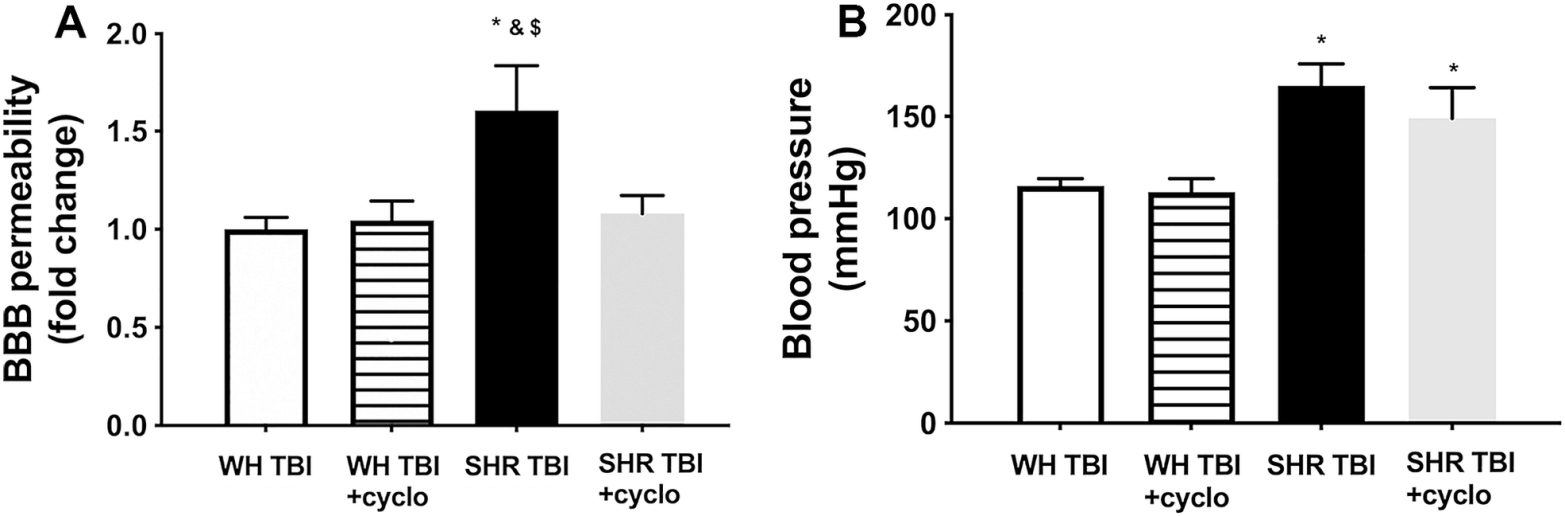
Mild traumatic brain injury induces persistent blood–brain barrier disruption in hypertensive rats, which is prevented by cyclosporine A treatment. **(A)** Blood–brain barrier permeability is shown by extravasated Evans blue content of cerebral tissue (fold change compared to control) in Wistar rats (WH TBI) and spontaneously hypertensive rats (SHR TBI) treated with cyclosporine A (+cyclo), that binds and inhibits intracellular cyclophilin A (15 min post-injury bolus dose: 20 mg/kg, the next 6 days: 10 mg/kg/day), or vehicle after mild traumatic brain injury (TBI). Data are mean ± S.E.M. (*n* = 8 in each group) ^*^p < 0.05 vs. Wistar TBI, ^&^*p* < 0.05 vs. Wistar TBI + cyclo, ^$^*p* < 0.05 vs. SHR TBI + cyclo. Panel **(B)** depicts blood pressure in Wistar rats (WH TBI) and spontaneously hypertensive rats (SHR TBI) treated with cyclosporine A after mild traumatic brain injury. Data are mean ± S.E.M. (*n* = 8 in each group) *p < 0.05 vs. Wistar TBI.

**Figure 2:**
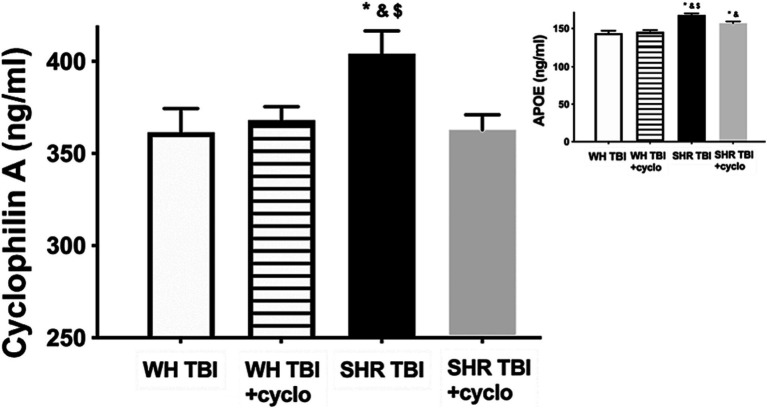
Mild traumatic brain injury increases cerebral cyclophilin A level, which is prevented by cyclosporine A treatment. The graph shows brain tissue levels of cyclophilin A (CyPA) in Wistar rats (WH TBI) and spontaneously hypertensive rats (SHR TBI) treated with cyclosporine A [depicted as +cyclo, binds and inhibits intracellular CyPA (15 min post-injury bolus dose: 20 mg/kg, the next 6 days: 10 mg/kg/day)] or vehicle after mild traumatic brain injury (TBI). Data are mean ± S.E.M. (*n* = 8 in each group) ^*^p < 0.05 vs. Wistar TBI, ^&^*p* < 0.05 vs. Wistar TBI + cyclo, ^$^*p* < 0.05 vs. SHR TBI + cyclo. Inlet shows apolipoprotein (ApoE) levels in brain homogenates of the same animals. Data are mean ± S.E.M. (*n* = 8 in each group) ^*^p < 0.05 vs. Wistar TBI, ^&^*p* < 0.05 vs. Wistar TBI + cyclo, ^$^*p* < 0.05 vs. SHR TBI + cyclo.

Mild traumatic brain injury led to significant (*p <* 0.05) disruption of the blood–brain barrier in hypertensive rats (*n* = 8) 7 days after trauma, as shown by increased Evans blue extravasation in these animals compared to normotensive controls (*n* = 8; Figure 1, OD620: WH + mTBI: 0.06 ± 0.002, WH + mTBI+cyclosporin:0.062 ± 0.001, SHR + mTBI: 0.096 ± 0.004, SHR + mTBI + cyclosporin: 0.064 ± 0.006). Importantly, BBB disruption was prevented in hypertensive rats by intraperitoneal cyclosporine treatment (Figure 2). Hypertensive rats had significantly (*p <* 0.05) elevated blood pressure, which was not affected by cyclosporine treatment (Figure 1). Non-toxic doses of intraperitoneal cyclosporine for 7 days (15 min post-injury bolus dose: 20 mg/kg, 10 mg/kg/day) effectively and specifically inhibits cyclophilin A in cerebral tissue (9). Accordingly, we found that mild traumatic brain injury led to significantly (*p <* 0.05) increased cyclophilin A levels [Figure 2; along with increased ApoE levels (Figure 2, inlet)] in cerebral tissue of spontaneously hypertensive rats (n = 8) 7 days after trauma compared to normotensive Wistar rats (*n* = 8; Figure 2, inlet), which were prevented by cyclosporine. This suggests that cyclophilin A plays a central role in mTBI-induced BBB disruption in hypertension.

### 3.2 Mild traumatic brain injury increases cerebral expression of NF-kB and activation of MMP-9 in hypertensive rats, which are prevented by cyclosporine a treatment

**Figure 3:**
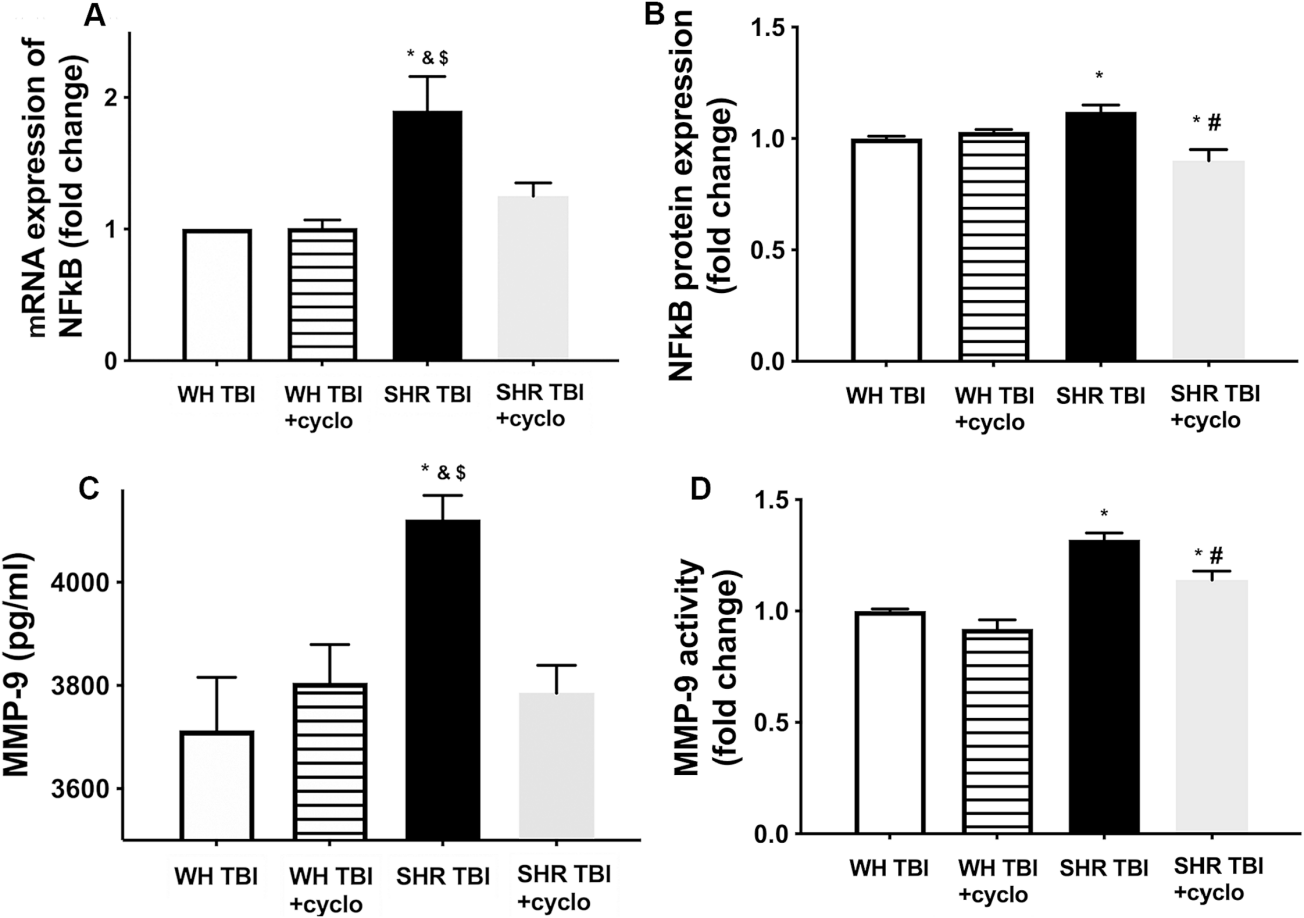
Mild traumatic brain injury induces increased cerebral expression of NF-kB and enhanced production and activation of MMP-9 in hypertensive rats, which are prevented by cyclosporine A treatment. **(A)** Expression of Nf-kB mRNA and protein **(B)** in brain tissue of Wistar rats (WH TBI) and spontaneously hypertensive rats (SHR TBI) treated with cyclosporine A (depicted as +cyclo) or vehicle after mild traumatic brain injury. Data are mean ± S.E.M. (*n* = 8 in each group) ^*^p < 0.05 vs. Wistar TBI, ^&^*p* < 0.05 vs. Wistar TBI + cyclo, ^$^*p* < 0.05 vs. SHR TBI + cyclo. **(C)** Cerebral matrix metalloproteinase 9 (MMP-9, pg./mL) levels and activity of the enzyme **(D)** in the same animals as shown on panel **(A,B)**. Data are mean ± S.E.M. (*n* = 8 in each group) ^*^p < 0.05 vs. Wistar TBI, ^&^*p* < 0.05 vs. Wistar TBI + cyclo, ^$^*p* < 0.05 vs. SHR TBI + cyclo.

We found that mTBI resulted in a significant (*p <* 0.05) increase in cerebral expression of NF-kB at both mRNA **(A)** and protein levels **(B)**, and induced production **(C)** and activation **(D)** of MMP-9 in cerebral tissue of hypertensive rats (*n* = 8) compared to control Wistar rats (*n* = 8) 7 days after trauma (Figure 3). It is of note that expression of NF-kB and MMP-9 and increased activity of MMP-9 were prevented by non-toxic doses of intraperitoneal cyclosporine for 7 days (15 min post-injury bolus dose: 20 mg/kg, the next 6 days: 10 mg/kg/day; Figure 3), suggesting that cyclophilin A led to BBB disruption (Figure 1) in hypertensive rats after mild brain trauma via the NF-kB-MMP-9 pathway.

### 3.3 Consequences of mild traumatic brain injury-induced BBB disruption in hypertensive rats: extravasation of blood components into the brain parenchyma and neuroinflammation

**Figure 4:**
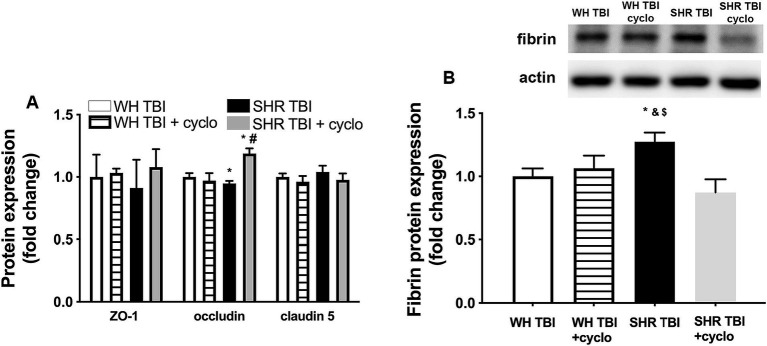
Mild traumatic brain injury induces extravasation of blood components into the brain parenchyma of hypertensive rats, which is prevented by cyclosporine A treatment. **(A)** Expression of blood–brain barrier proteins ZO-1, occludin and claudin 5 in cerebral tissue of Wistar rats (WH TBI) and spontaneously hypertensive rats (SHR TBI) treated with cyclosporine A (depicted as +cyclo), that binds and inhibits intracellular cyclophilin A (15 min post-injury bolus dose: 20 mg/kg, the next 6 days: 10 mg/kg/day) or vehicle after mild traumatic brain injury (TBI). Data are mean ± S.E.M. (*n* = 8 in each group) *p < 0.05 vs. Wistar TBI, #*p* < 0.05 vs. SHR TBI. **(B)** Summary data depicts cerebral fibrin protein level in cortical tissue of Wistar rats (WH TBI) and spontaneously hypertensive rats (SHR TBI) treated with cyclosporine A (depicted as +cyclo), that binds and inhibits intracellular cyclophilin A (15 min post-injury bolus dose: 20 mg/kg, the next 6 days: 10 mg/kg/day) or vehicle after mild traumatic brain injury (TBI). Data are mean ± S.E.M. (*n* = 8 in each group) ^*^p < 0.05 vs. Wistar TBI, ^&^*p* < 0.05 vs. Wistar TBI + cyclo, ^$^*p* < 0.05 vs. SHR TBI + cyclo. A representative blot presents fibrin level in perfused cerebral tissue of one animal from each animal group on panel **(A)**.

**Figure 5:**
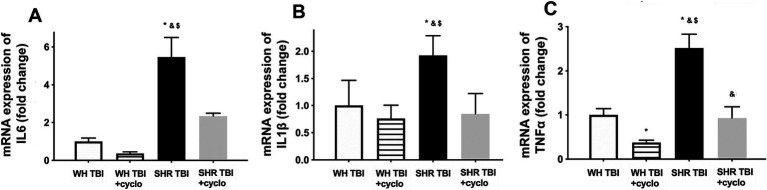
Mild traumatic brain injury induces persistent neuroinflammation in the brain parenchyma of hypertensive rats, which is prevented by cyclosporine A treatment. mRNA expression of inflammatory cytokines IL-6 **(A)**, IL-1β **(B),** and TNFα **(C)** in cortical tissue of Wistar rats (WH TBI) and spontaneously hypertensive rats (SHR TBI) treated with cyclosporine A (depicted as +cyclo), that binds and inhibits intracellular cyclophilin A (15 min post-injury bolus dose: 20 mg/kg, the next 6 days: 10 mg/kg/day) or vehicle after mild traumatic brain injury (TBI). Data are means ± S.E.M. (*n* = 8 in each group) ^*^p < 0.05 vs. Wistar TBI, ^&^*p* < 0.05 vs. Wistar TBI + cyclo, ^$^*p* < 0.05 vs. SHR TBI + cyclo.

We observed that in line with mild TBI-induced BBB disruption in hypertensive rats, cerebral expression of blood–brain barrier protein ZO-1 was decreased in hypertensive rats 7 days after mild brain trauma compared to normotensive rats with and without mTBI. Attenuated ZO-1 expression was prevented by non-toxic doses of intraperitoneal cyclosporine for 7 days (Figure 4A). mTBI and hypertension-induced changes in the expression of occludin and claudin 5 in cerebral tissue of normotensive and hypertensive rats with and without mTBI did not reach statistical significance (Figure 4A). Structural damage of the BBB in hypertensive rats after mild TBI most likely allows blood components to enter the brain parenchyma, suggested by the significantly (*p <* 0.05) increased fibrin content of cortical tissue of spontaneously hypertensive rats (n = 8) after mTBI compared to control normotensive Wistar rats (*n* = 8; Figure 4B). Fibrin accumulation was associated with significantly (*p <* 0.05) increased level of the inflammatory cytokines IL-1β, IL-6, and TNFα in cortical tissue of spontaneously hypertensive rats after mild TBI compared to normotensive animals (Figure 5), suggesting that extravasation of serum components lead to persistent neuroinflammation in SHRs after mild TBI. Importantly, both fibrin extravasation (Figure 4) and persistent neuroinflammation (Figure 5) were prevented in SHRs after mTBI by non-toxic doses of intraperitoneal cyclosporine for 7 days.

## 4 Discussion

Mild traumatic brain injury is about 80% of brain trauma cases (15), and its pathophysiological consequences can lead to long-term cognitive impairment in mTBI patients (16). It was shown that mTBI induces BBB disruption in preclinical models and patients, as well, contributing to neurodegeneration and cognitive dysfunction developing after the injury (17–19). mTBI affects specifically elder patients with several possible comorbidities, among which hypertension is the most frequent one. Cerebrovascular risk factors, such as hypertension were also shown to impair BBB function (20). We recently demonstrated that mTBI induced persistent disruption of the BBB in hypertensive rats compared to normotensive control animals, which was associated with neuroinflammation and cognitive decline (5), but the underlying mechanisms remained elusive. Cyclophilin A, an intracellular immunophilin, which can be secreted in response to various stimuli (21–23) is produced by the cells of the neurovascular unit (9), and was shown to be elevated in the sera of untreated hypertensive patients (7), as well as after severe TBI, and emerged as a predictor for mortality and outcome of these patients (6). In line with these findings CyPA level was significantly increased in isolated cerebral microvessels of rats after severe TBI, and administration of cyclosporine A, a blocker of CyPA production inhibited BBB disruption (8). Also, cyclosporine treatment enhanced BBB repair by decreasing CyPA activity in mice (24). Here we demonstrate that mTBI induced long term BBB disruption (Figure 1) and increased cerebral CyPA levels (Figure 2) in hypertensive rats, which were prevented by cyclosporin treatment (Figures 1, 2). These results and the studies mentioned above strongly suggest that mTBI and hypertension interact to promote cerebral CyPA production, which leads to BBB disruption. It has to be noted that cyclosporin was shown to decrease transendothelial electrical resistance in an *in vitro* cell culture model of BBB (25). Future studies should explain the conflicting *in vitro* and *in vivo* results regarding BBB function after cyclosporin treatment.

The mechanisms how mTBI and hypertension synergize to promote CyPA production are likely multifaceted. Both TBI and hypertension were shown to activate astrocytes and to induce mitochondrial oxidative stress, which can be a stimulus for cyclophilin production (21–23). Also, Apolipoprotein E4 activates cyclophilin production in pericytes (9), and was shown to be produced in astrocytes after severe TBI in mice, leading to BBB disruption (24, 26). The role of ApoE in mTBI and hypertension-induced increased cerebral cyclophilin production is also suggested by our results, demonstrating that ApoE is significantly increased in cerebral tissue of hypertensive rats after mTBI, compared to hypertensive sham operated and normotensive rats with and without mTBI (Figure 2). The exact cellular source and isoform of mTBI-induced ApoE in hypertension should be established by future studies.

The ApoE-cyclophilin pathway was shown (9) to activate NF-kB in pericytes, which transcriptionally activates the gelatinase MMP-9 in cerebral vessels, leading to degradation of capillary basement membrane and tight junction proteins and ultimately BBB disruption (9, 27–29). Importantly, after severe TBI astrocyte-derived ApoE4 induced BBB disruption via the activation of the NF-kB-MMP-9 pathway (10, 24, 26). We demonstrate here that mTBI leads to a significant increase in cerebral NF-kB expression, enhanced production and activation of MMP-9 and attenuated expression of the BBB protein ZO-1 in hypertension (Figures 3, 4), which were prevented by cyclosporine treatment. This suggests that mTBI and hypertension-induced enhanced cyclophilin A leads to BBB breakdown via the NF-kB-MMP-9 pathway. It was mentioned above that the elderly are specifically prone to suffer mTBI, and they exhibit the highest prevalence of hypertension in the population. From this point it is significant that aging is associated with increasing ApoE levels (30), it leads to increased NF-kB activity in cerebral vessels (31), and aging and hypertension interact to promote increased MMP-9 activity and formation of cerebral microbleeds (32). Further studies should establish the effect of mTBI on the ApoE-cyclophilin-NF-kB-MMP-9 pathway and BBB function in aging and hypertension.

mTBI-induced BBB breakdown most likely has detrimental consequences on cerebral tissue and brain function. Extending our previous results (5) we show here that mTBI resulted in accumulation of fibrin in cerebral tissue of hypertensive rats (Figure 3) and a significant increase in the cerebral production of inflammatory cytokines IL-1β, IL-6 and TNFα (Figure 5), which were prevented by protecting the BBB via inhibiting cyclophilin production. Previous studies suggested that accumulation of blood borne substances in the brain parenchyma is the link between BBB disruption and neuronal damage. For instance, in pericyte-deficient mice deposition of fibrinogen in brain parenchyma (33) through the disrupted BBB was associated with activation and increasing number of microglia, production of inflammatory cytokines and oxidative stress (34), leading to further activation of proteases (35), probably producing a positive feedback loop and perpetuating the pathological process. Cyclosporine treatment may also have a direct effect on mTBI and hypertension-induced neuroinflammatory response in cerebral tissue, which should be studied in the future. Previous studies showed that chronic neuroinflammation in hippocampi of mice led to structural modification of axons and dendrites of neurons, and dysregulated genes involved in regulation of normal synaptic plasticity and neuronal function (such as Bdnf, Homer1, and Dlg4) (36, 37). Supporting the role of BBB disruption-related neuroinflammation in the development of cognitive dysfunction we also demonstrated that mTBI-induced persistent neuroinflammation in hypertension was associated with cognitive decline (5). Future studies should verify on a larger number of animals whether prevention of mTBI-induced disruption of the BBB in hypertension would also prevent the development of cognitive decline. It also has to be noted here and studied in the future, that BBB-disruption may lead to edema of cerebral tissue, which most likely impairs capillary blood flow and therefore neuronal function.

In conclusion, we show that mild TBI and hypertension interact to promote disruption of the blood–brain barrier which can be prevented by blocking the CyPA-NF-kB-MMP-9 pathway. Based on our results we propose that hypertensive (especially elder) patients should be assessed differently than normotensive patients (by quantifying BBB function, cognitive function etc.) after mTBI and clinically safe cyclosporine treatment may be beneficial in preventing the long-term consequences of mild brain trauma.

## Data availability statement

The raw data supporting the conclusions of this article will be made available by the authors, without undue reservation.

## Ethics statement

The animal study was approved by Animal Welfare Committee, University of Pecs, and the National Scientific Ethical Committee on Animal Experimentation, Hungary (nr: BA02/2000-35/2022) and carried out in accordance with the ARRIVE guidelines. The study was conducted in accordance with the local legislation and institutional requirements.

## Author contributions

DL-E and PT wrote the original draft. PT and EC were responsible for the conceptualization. The manuscript was reviewed and edited by PT, ZM-S, EH, NS, BF, and KA. Supervision was carried out by AB and ZU. Project administration was assigned to DL-E. Funding acquisition was conducted by PT and ZU. All authors contributed to the article and approved the submitted version.
